# Decolorization of Lignin for High‐Resolution 3D Printing of High Lignin‐Content Composites

**DOI:** 10.1002/advs.202406311

**Published:** 2024-08-13

**Authors:** David Böcherer, Ramin Montazeri, Yuanyuan Li, Silvio Tisato, Leonhard Hambitzer, Dorothea Helmer

**Affiliations:** ^1^ Department of Microsystems Engineering University of Freiburg 79110 Freiburg Germany; ^2^ Freiburg Materials Research Center (FMF) University of Freiburg 79104 Freiburg Germany

**Keywords:** decolorization of lignin, high‐resolution 3D printing, lignin biocomposites

## Abstract

Lignin, one of the most abundant biomaterials and a large‐scale industrial waste product, is a promising filler for polymers as it reduces the amount of fossil resources and is readily available. 3D printing is well‐known for producing detailed polymer structures in small sizes at low waste production. Especially light‐assisted 3D printing is a powerful technique for production of high‐resolution structures. However, lignin acts as a very efficient absorber for UV and visible light limiting the printability of lignin composites, reducing its potential as a high‐volume filler. In this work, the decolorization of lignin is presented for high‐resolution 3D printing of biocomposites with lignin content up to 40 wt.%. Organosolv lignin (OSL) is decolorized by an optimized low‐energy process of acetylation and subsequent UV irradiation reducing the UV absorbance by 71%. By integration of decolorized lignin into bio‐based tetrahydrofurfuryl acrylate (THFA), a lignin content of 40 wt.% and a resolution of 250 µm is achieved. Due to the reinforcing properties of lignin, the stiffness and strength of the material is increased by factors of 15 and 2.3, respectively. This work paves the way for the re‐use of a large amount of lignin waste for 3D printing of tough materials at high resolution.

## Graphical abstract

To enable the integration of lignin at high content for the high‐resolution 3D printing of sustainable materials, lignin is decolorized by UV irradiation. The decolorized lignin is integrated in bio‐based tetrahydrofurfuryl acrylate to obtain a photocurable resin. Using a commercial DLP printer lignin composites with a lignin content up to 40 wt.% and a resolution of 250 µm are fabricated.

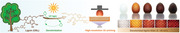

## 1 Introduction

In the last decade, the development of novel biocomposite materials to promote the fabrication of more renewable and sustainable materials has received increasing attention. Lignin as the second most abundant biomaterial on earth and waste material of the paper industry is a promising component for the integration in biocomposite materials.^[^ ^1^ ^]^ Lignin has been integrated as a filler with a content of up to 70 wt.% in many different composite materials based on polyvinyl alcohol,^[^ ^2^, ^3^, ^4^ ^]^ polypropylene,^[^ ^5^, ^6^, ^7^ ^]^ and polyethylene.^[^ ^8^, ^9^, ^10^, ^11^ ^]^ Most of these thermoplastic composite materials are fabricated by mold‐based processing methods, which are industrially relevant, however they limit the flexibility in design of the produced materials.^[^ ^12^ ^]^ 3D printing is well‐known for the high flexibility in design as well as the low waste production of the process.^[^ ^13^ ^]^ 3D printing of lignin composites enables customized fabrication of lignin composite materials and expands their field of application. Using extrusion‐based 3D printing methods, lignin can be integrated in very high content of 20–60 wt.% into various thermoplastic materials such as polylactic acid (PLA), polyhydroxy butyrate (PHB), Pluronic and hydroxypropyl cellulose.^[^ ^14^, ^15^, ^16^, ^17^ ^]^ Recently, fused deposition modeling (FDM) of high‐performance lignin composites by incorporation of up to 60 wt.% of lignin in Nylon12 was shown, increasing the stiffness of the material by 70% to 3.0 GPa.^[^ ^18^ ^]^ Direct ink writing (DIW) was used to 3D print fully bio‐based composites in hydroxypropyl cellulose with 50 wt.% of lignin.^[^ ^19^ ^]^ However, these extrusion‐based printing methods are limited in the resolution of the printed objects and thus are not suitable for the fabrication of high‐precision structures. For higher precision, light‐induced 3D printing techniques such as stereolithography (SLA) or digital light processing (DLP) are favored. These 3D printing technologies allow the individual structuring of lignin biocomposites with detailed features. The high‐resolution DLP printing in the submillimeter scale of biocomposites with a lignin content of 0.5–2.0 wt.% was achieved by the addition of lignin nanoparticles to a vanillin‐ and eugenol‐based photo resin.^[^ ^20^ ^]^ Even with a low filler content of only 1–5 wt.%, the 3D printed lignin composites showed an increased strength in mechanical properties.^[^ ^21^, ^22^ ^]^ Due to the optical properties of lignin with very high absorption of UV and visible light, light‐induced 3D printing of lignin composites with filler content higher than 5 wt.% is challenging. Recently, up to 15 wt.% of acrylated lignin was integrated in a mixture of commercial resins for the successful 3D printing with resolution in the millimeter scale.^[^ ^23^ ^]^ By grafting of propylene oxide onto the lignin molecule, Keck et al. developed a liquid prepolymer resin for 3D printing with lignin content of 33 wt.%.^[^ ^24^ ^]^ However, the black color of the photo resin limited the printing resolution to the millimeter scale and the design to simple solid structures. To allow high‐resolution 3D printing of lignin composites with a high lignin content of >10 wt.% in the submillimeter scale, the lignin needs to be modified for reduced light absorption. As the dark color of lignin limits its usage for many applications, various procedures have been developed to reduce the color of lignin such as fractionated separation,^[^ ^25^, ^26^ ^]^ oxidation,^[^ ^27^, ^28^, ^29^, ^30^ ^]^ or chemical modification.^[^ ^31^ ^]^ By fractionated separation of low molecular weight lignin by methanol/water extraction^[^ ^25^ ^]^ or lowering pH^[^ ^26^ ^]^ lignin with light brown color was separated. Harsh conditions such as boiling or UV irradiation in water were used for the fabrication of transparent wood^[^ ^28^ ^]^ or faded lignin.^[^ ^27^ ^]^ Recently, acetylation of lignin was used to reduce the color intensity of lignin and to increase its compatibility in PLA.^[^ ^32^ ^]^ The acetylated lignin/PLA composites with up to 10 wt.% of filler showed increased transparency (76%) for 30 µm thin foils compared to the pristine lignin‐PLA composite material (11%). Besides the harsh treatment with oxidizing agents, chemical modification or fractionated separation, also a more sustainable treatment of UV irradiation was shown to reduce the coloration of lignin.^[^ ^33^ ^]^ Lignin was dissolved in tetrahydrofuran (THF) and irradiated with UV light (365 nm) resulting in decreased absorbance. For the 3D printing of high lignin‐content composites, a suitable bio‐based photo resin as matrix material is required. In recent years, various bio‐based photo resins such as (meth)acrylated modifications of vegetable oils, isoborneol and tetrahydrofuran were developed.^[^ ^34^ ^]^ Tetrahydrofurfuryl acrylate (THFA) was used in various photo resins such as for the polymerization of renewable and functional latexes.^[^ ^35^ ^]^ Moreover, THF‐based photo resins were developed for the wavelength selective multi‐material 3D printing^[^ ^36^ ^]^ as well as SLA printing of reprocessable acrylate vitrimers.^[^ ^37^ ^]^ As lignin shows a good solubility in THF and THFA was proven to be a suitable monomer for photo‐initiated 3D printing techniques, THFA can be a promising matrix material for the DLP printing of lignin composite materials.

In this work, we show for the first time the high‐resolution vat polymerization 3D printing of lignin composite materials with high lignin content up to 40 wt.%. The dark brown lignin is decolorized by UV irradiation to reduce its light‐absorbing properties and make it suitable as a filler for high‐resolution vat polymerization 3D printing. Using a commercial DLP 3D printer, biocomposite materials with lignin content up to 40 wt.% are printed with a resolution down to 250 µm. Besides the high‐precision printing, the integration of lignin as a reinforcing agent also improves the mechanical properties of the composite material. The addition of 40 wt.% decolorized lignin leads to increased stiffness and strength by factors of 15 and 2.3, respectively. Thus, the decolorization of lignin by UV irradiation enables the re‐use of lignin for high‐resolution 3D printing of tough biocomposites with great potential to reduce the environmental footprint of 3D printing.

## 2 Results and Discussion

### 2.1 Decolorization of Lignin for 3D Printing of High Lignin‐Content Composites

**Figure 1:**
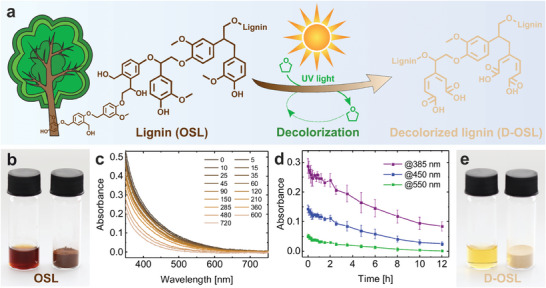
Decolorization process of organosolv lignin (OSL) by UV irradiation. a) Schematic illustration of the decolorization process of dark brown OSL to decolorized lignin (D‐OSL) by UV irradiation in recyclable THF. b) Optical appearance of pristine OSL in THF (2.5 g L^−1^) for decolorization (left) and in dry‐state (right). c) UV/Vis spectra of solutions of OSL in THF during the decolorization process with irradiation times from 0 to 12 h using a 14.4 W UV LED. The legend indicates the irradiation time in minutes d) Absorbance of the solution of OSL in THF during the decolorization process at wavelengths of 385, 450, and 550 nm depending on irradiation times from 0 to 12 h showing the drastically decreasing absorption properties of D‐OSL. e) Optical appearance of D‐OSL in THF (2.5 g L^−1^) after decolorization (left) and in dry‐state (right).

Its strong UV absorption limits the use of lignin as a sustainable reinforcing filler for high‐resolution structuring by 3D printing. To enable the vat polymerization 3D printing of composites with high lignin content, these strong UV absorption properties of lignin need to be reduced. Wang et al. introduced a decolorization process of lignin by UV irradiation in THF.^[^ ^33^ ^]^ Here, this method was optimized by acetylation of the lignin as a pre‐modification step. The additional optimization of concentration, volume, and UV irradiation intensity enabled a low‐energy method with improved efficiency for the decolorization of lignin (see **Figure** **1**a). In the first step, the organosolv lignin (OSL) was acetylated by reaction with acetic anhydride in pyridine to increase its solubility in THF. In a second step the decolorization process by UV irradiation of acetylated OSL (A‐OSL) in THF was carried out. Instead of an irradiation for 200 h with a 200 W cold cathode UV lamp to produce 2 g of decolorized lignin, our optimized method applied a 14.4 W UV LED array for 12 h to a solution of 1.25 g of A‐OSL in 0.5 L of THF to produce the decolorized lignin (D‐OSL) faster and more efficient. Moreover, the THF used for the decolorization process was recycled by rotary evaporation and used again for the next decolorization run to increase the sustainability of the process. The NMR spectra of the THF before and after UV irradiation for 24 h in Figure S1 (Supporting Information) show that there is no change in the solvent by the process and it can be recycled and reused enhancing the sustainability of the process. As can be seen in the Figure 1b–e the dark brown color of the original OSL is drastically reduced to a light brownish color of the D‐OSL. In Figure 1c the decolorization process of acetylated OSL to decolorized OSL is analyzed by UV/Vis analysis. The change in absorbance spectra over time clearly shows the continuous decolorization of the lignin by UV irradiation. The quantitative analysis in Figure 1d demonstrates that the absorbance of the D‐OSL decreased by 71%, 82%, and 98% at the wavelengths of 385, 450, and 550 nm, respectively. Thus, besides the reduced irradiation time and power, the modified method led to a higher decolorization degree of 82% at 450 nm compared to the decolorization degree of 65% in the original method.^[^ ^33^ ^]^ This proves the successful optimization of the decolorization process by acetylation as pre‐modification as well as optimized concentration, volume, and UV irradiation intensity for a more efficient decolorization of lignin.

### 2.2 Resin Development for High‐Resolution 3D Printing of High Lignin‐Content Composites

To develop a bio‐based resin with high lignin‐content for high‐resolution 3D printing, a matrix material system with suitable reactivity, viscosity and compatibility to lignin was designed. Lignin shows very low solubility in most organic solvents or monomers – leading to inhomogeneous mixing, fast sedimentation of the lignin within the resin and low printing resolution. As THF showed good compatibility with lignin and was able to dissolve a high amount of lignin, THF‐based acrylate species were chosen as a promising material for resin design. Here, tetrahydrofurfuryl acrylate (THFA) was chosen due to its low viscosity and bio‐based origin from hemicellulose. In solubility tests, homogeneous solutions of D‐OSL in THFA with lignin content up to 50 wt.% were obtained. However, the low reactivity of THFA upon photoinitiated radical polymerization caused by its mono functionality inhibited a fast and successful 3D printing process in high‐resolution. Moreover, the weak mechanical properties of the poly(tetrahydrofurfuryl acrylate) (pTHFA) based on its thermoplastic behavior with a glass transition temperature of −11 °C^[^ ^38^ ^]^ prevented the printing of stable 3D structures and detailed features. To overcome these problems of low reactivity and weak mechanical properties, the tetrafunctional monomer pentaerythritol tetraacrylate (PETA) was added to the matrix material system. A basic reference resin DL‐0 consisting of 87 wt.% THFA and 13 wt.% PETA containing initator 2,4,6‐trimethylbenzoyl diphenyl phosphine oxide (TPO) and absorber Tinuvin 326 was developed. For preparation of the lignin composites, the matrix material of THFA and PETA (87:13, w:w) was mixed with different content of lignin of 10%, 20%, 30% and 40%. As the lignin still maintained a strong UV‐absorption, the absorber Tinuvin 326 was not required for all resins containing lignin. The detailed compositions of the resins are listed in **Table** **1**.

**Table 1:** 3D printing resin compositions containing decolorized lignin fillers (D‐OSL) from 0 wt.% to 40 wt.%. Resins contain monoacrylate THFA as matrix and tetraacrylate PETA as crosslinker, Tinuvin 326 as an absorber, and TPO as a photoinitiator. All compositions are based on the reference resin DL‐0, in which the D‐OSL is dissolved. As D‐OSL is a strong absorber, the resins containing D‐OSL did not require additional absorber Tinuvin 326.

Name	D‐OSL [wt.%]	THFA [wt.%]	PETA [wt.%]	Tinuvin 326^a)^ [wt.%]	TPO^a)^ [wt.%]
DL‐0	0.0	87.0	13.0	1.0	2.0
DL‐10	10.0	78.3	11.7	0.0	2.0
DL‐20	20.0	69.6	10.4	0.0	2.0
DL‐30	30.0	60.9	9.1	0.0	2.0
DL‐40	40.0	52.2	7.8	0.0	2.0

^a)^ Amount of TPO and Tinuvin 326 was determined related to the total mass of D‐OSL, THFA and PETA.

**Figure 2:**
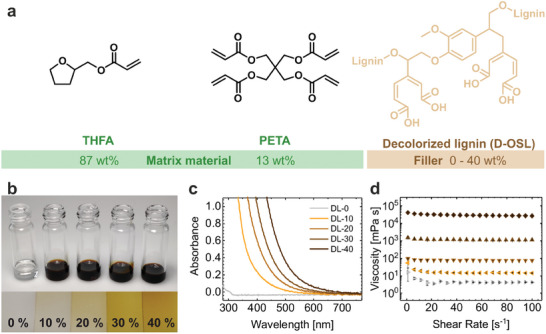
Composition, optical properties, and viscosity of the developed 3D printing resins containing decolorized lignin. a) Resin composition of lignin composite materials with THFA as matrix, PETA as crosslinker and decolorized lignin as bio‐based filler. b) Optical appearance of the mixed resins with lignin contents from 0 wt.% to 40 wt.% in glass vials and as thin films. The bottom picture shows the thin films of the resins of 40 µm thickness between two glass slides. c) Absorbance spectra of the individual resins with lignin contents from 0 wt.% to 40 wt.%. Absorbance was measured for the thin resin films shown in b). Increased lignin content leads to stronger absorption in the UV and blue‐to‐green light range. d) Shear rate‐dependent viscosity measurements of the individual resins with lignin contents from 0 wt.% to 40 wt.% showing an increase in viscosity for higher lignin content. The legend in (c) also applies for (d).

**Figure** **2**a, visualizes the optimal acrylate composition (THFA:PETA = 87:13, wt:wt) and the varying solid loading of up to 40 wt.% of D‐OSL. In Figure 2b, the optical appearance of the resins is shown in the glass vials and as thin films between two glass slides. An increase in absorption with higher lignin content can be clearly observed. The image also shows that even at the lowest lignin content of 10 wt.% the resin in the glass vial is very dark demonstrating the challenge for 3D printing of strong absorption properties of the resin. The thin film images (film thickness of 40 µm) of the individual resins show that reducing the layer thickness leads to suitable transparency and light penetration for 3D printing. The influence of the lignin content on the transparency of the individual resins was quantified by UV/Vis measurements. In Figure 2c, the absorbance of the 40 µm films of the lignin resins is presented showing the increased absorption at higher lignin content. In comparison, the optical properties of the same resins with untreated organosolv lignin (OSL) instead of decolorized lignin showed significantly stronger absorption properties (Figure S2, Supporting Information). An OSL content of only 10 wt.% led to a similar UV/vis absorption as the integration of 40 wt.% of decolorized lignin demonstrating the great potential of the decolorization of lignin for integration of lignin at high content. Besides optical properties, the viscosity of the resins plays an important role for the 3D printing process. As demonstrated in Figure 2d, the viscosity of the resins increases from 4 mPas for the reference material without any lignin content to 14, 77, 1085, and 26 013 mPas for resins with lignin content of 10 wt.%, 20 wt.%, 30 wt.%, and 40 wt.%, respectively. Common vat polymerization systems are limited to a resin viscosity of ≈5000 mPas,^[^ ^39^ ^]^ and first tests showed that the high viscosity of the DL‐40 resin prevents it from being effectively printed. To overcome this problem, the viscosity of the resin was reduced by increasing the printing temperature to 40 °C. As can be seen in Figure S3 (Supporting Information), the viscosity of the DL‐40 resin was reduced by 82% to 4763 mPas at 40 °C. This way, DL‐40 resins with 40 wt.% lignin content could be used for vat polymerization 3D printing.

### 2.3 High‐Resolution 3D Printing of High Lignin‐Content Composites

**Figure 3:**
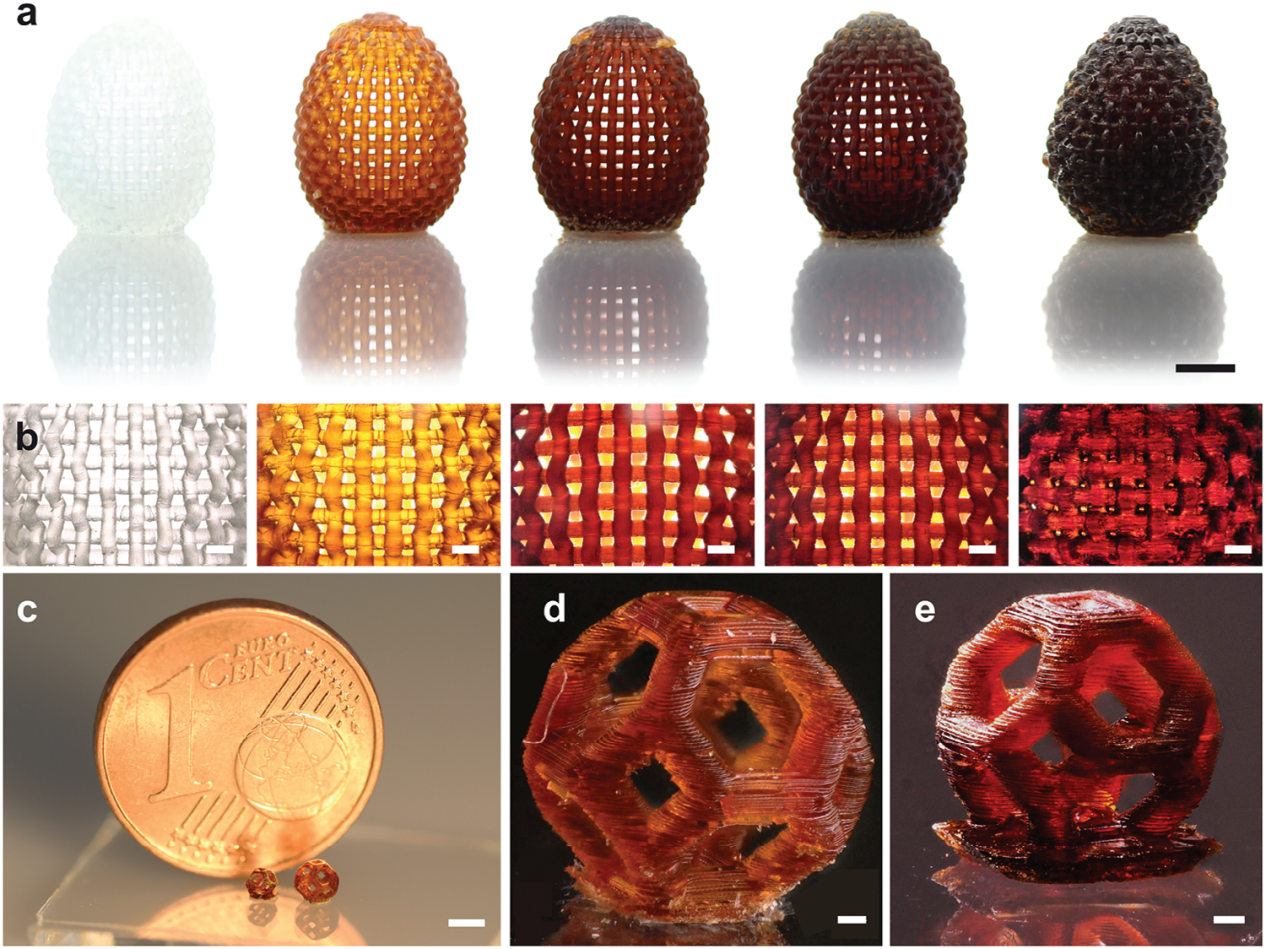
3D printed structures of individual lignin composites. a) 3D printed lattice eggs of DL‐0, DL‐10, DL‐20, DL‐30, and DL‐40 (from left to right) containing 0 wt.% to 40 wt.% of lignin. The prints of the lattice eggs in the size of 2 cm with line width and hole diameter of ≈0.8 mm show the good printability of the developed lignin composites. The design of the lattice eggs was adopted from external source.^[^ ^40^ ^]^ b) Microscopy images of the detailed lattice structure of the lattice eggs. The lattice eggs of DL‐0 to DL‐30 show holes in the size of 800 µm. The lines of the DL‐40 lattice egg show a broadening effect leading to holes in the size of 200–300 µm. c) 3D printed truncated octahedrons of DL‐30 (right) and DL‐40 (left) in the size of 2.5 and 1.6 mm, respectively. d) Microscopy image of the DL‐30 truncated octahedron with a line width of 300 µm. e) Microscopy image of the DL‐40 octahedron with a line width of 250 µm. These 3D printed structures in the size of millimeters with feature sizes of a few hundreds of microns demonstrate the great potential of high‐resolution printing of lignin composites. Scale bars: a) 5, b) 1, c) 2, d) 200, e) 200 µm.

The strong absorption properties of conventional lignin materials reduce the printability and resolution of the 3D printing process for high lignin content. To structure our decolorized lignin composite materials with reduced absorption properties and allow the fabrication of detailed objects in the submillimeter scale for advanced applications, a DLP 3D printing process was optimized for high‐resolution structuring of high lignin‐content composites. The printing parameters were optimized regarding the absorption properties, reactivity and viscosity of the developed lignin resins. In addition to the absorption measurements, illumination time‐dependent curing depth measurements were done to test the reactivity and light penetration behavior of the resins. As can be seen in Figure S4a (Supporting Information), increasing the decolorized lignin content from 10 wt.% to 40 wt.% drastically decreased the curing depth of the material due to the strong UV absorption. However, compared to the curing curves of the reference resins with original OSL (10 wt.%) and acetylated OSL (10 wt.%) as filler (see Figure S4b, Supporting Information), the use of decolorized lignin significantly improved the curing depth and therefore enabled high‐resolution 3D printing of composites with high lignin content. Based on the curing curves, the printing parameters presented in Table S1 (Supporting Information) were found to be the optimal. While all materials with decolorized lignin content from 0 wt.% to 30 wt.% could be printed at room temperature of 25 °C, the material DL‐40 with 40 wt.% of decolorized lignin was printed at 40 °C to reduce its high viscosity and allow sufficient flow‐back of the resin after each printed layer. The high light intensity of 29.3 mWcm^−2^, the small layer thickness of 25 µm, and the increased exposure and burn‐in exposure times allowed for an adequate curing process to structure each individual resin by high‐resolution 3D printing. The successful printing of each material with decolorized lignin content up to 40 wt.% is demonstrated in **Figure** **3**.

The complex structures of the lattice eggs in the size of 2 cm were printed for each material including 0 wt.% to 40 wt.% of decolorized lignin, showing the good printability of the developed materials. As shown in the microscopy pictures in Figure 3b, the lattice eggs of the samples DL‐0 to DL‐30 showed lines with a width of 700 µm and holes in the size of 700 µm, while the lattice egg of the DL‐40 material showed lines with a width of 1 mm and smaller holes in the size of 200–400 µm. The broadening of the printed lines can be attributed to the increasing viscosity over the long printing time of 14 h at elevated temperature (see Figure S3, Supporting Information). This effect is also in compliance with the successful high‐resolution 3D printing of the DL‐30 and DL‐40 materials in smaller size and shorter printing times. In Figure 3c, the high‐resolution 3D printing of the resins with high lignin loading of 30 wt.% and 40 wt.% is demonstrated. Truncated octahedrons in the size of 2.5 mm and 1.6 mm were printed successfully from the DL‐30 and DL‐40 resins. The optical microscopy pictures in Figure 3d,e show the high precision of the prints with line widths in the size of 300 and 250 µm, respectively. To determine the printability of the individual lignin materials with lignin content from 0 to 40 wt.%, all materials were printed in a test design for printability quantification. As can be seen in Figure S5 (Supporting Information), for all resins with lignin content up to 40 wt.% small bars and channels in size of 100 µm were printed successfully. Developing of the high‐viscosity resin DL‐40 was challenging and caused some roughness on the print surface as observed in Figure S5 (Supporting Information). These 3D printed structures in the size of millimeters with feature sizes of a few hundreds of microns demonstrate the great potential of the first high‐resolution printing of lignin composites up to 40 wt.% of lignin filler. Thus, our high lignin‐content composites are suitable for 3D printing of structures in the size of centimeters as well as for structuring of features down to 250 µm providing a sustainable material for a wide range of applications. To provide a brief idea of the climate impact properties of our lignin composites, we determined the bio renewable carbon content (BRC) of the materials ranging from 54% (DL‐0) to 66% (DL‐40). This shows that the integration of lignin at high content in a bio‐based matrix material allows for the fabrication of materials with 66% renewable character.

### 2.4 Material Properties of High Lignin‐Content Composites

**Figure 4:**
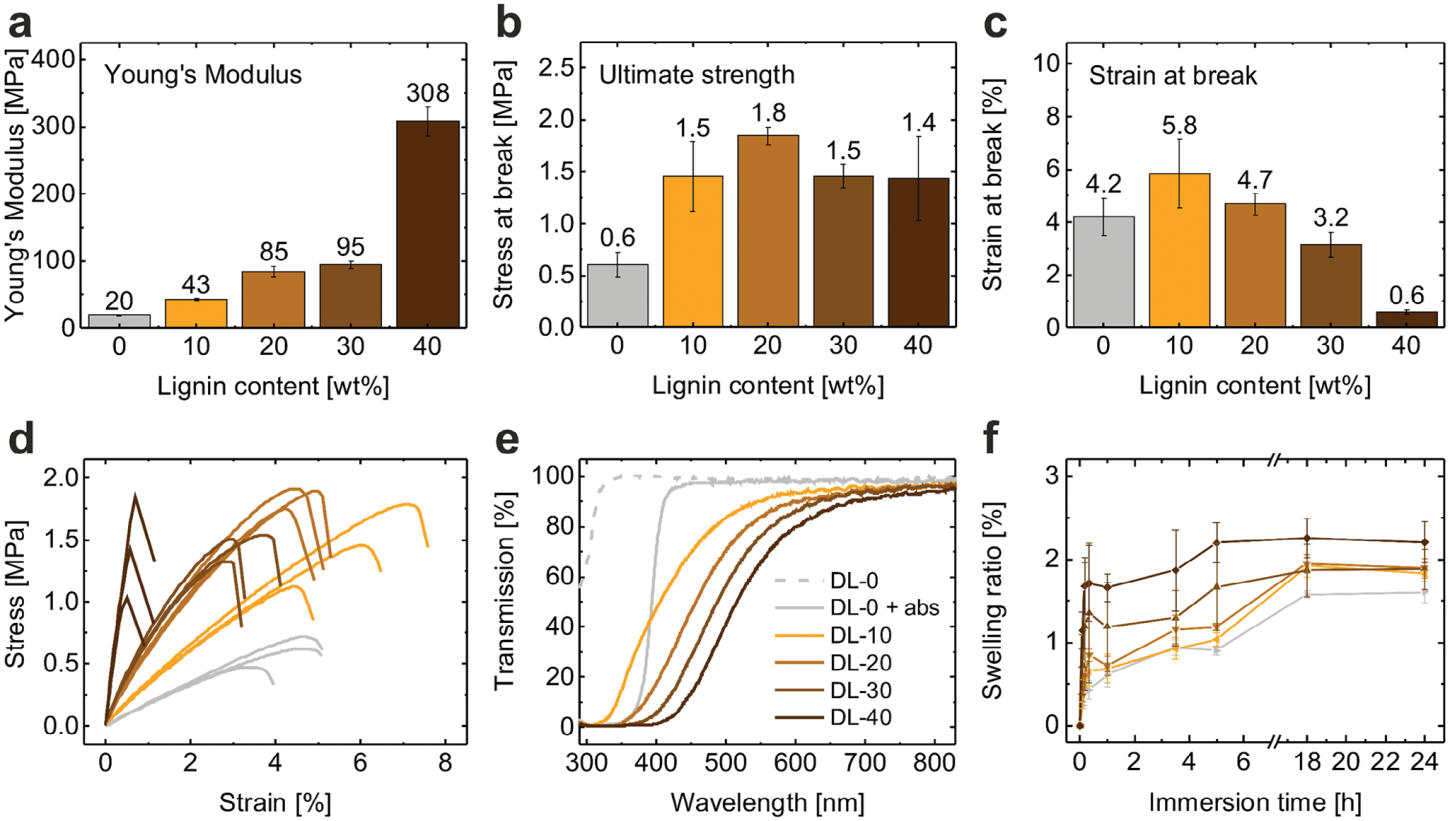
Material properties of lignin composite materials with lignin content of 0 wt.% to 40 wt.%. a) Young's Modulus of lignin materials showing a drastic increase in stiffness with higher lignin content. b) Ultimate stress of lignin materials depending on lignin content with a maximum ultimate stress for 20 wt.% of lignin. c) Strain at break of lignin materials depending on lignin content with a maximum strain at break for 10 wt.% of lignin. d) Complete tensile testing curves with three measurements of each material for determination of Young's Modulus, ultimate stress, and strain at break. e) UV/Vis analysis of thin films (40 µm) for characterization of optical properties of lignin composite materials. As the reference material DL‐0 needs additional absorber Tinuvin for 3D printing, the optical properties of the material are presented without Tinuvin (DL‐0) and with Tinuvin (DL‐0 + abs). f) Swelling test of lignin composite materials showing a low swelling ratio (gain in weight) of <2.5% for all materials with up to 40 wt.% of lignin content. The legend in (e) also applies for (d) and (f).

The material properties of the developed resins are of high interest to ensure high performance for both everyday use and advanced applications. As lignin is well‐known for its impact as reinforcing filler for various materials, the mechanical performance of our developed 3D printed lignin materials was tested. To analyze the impact of the integrated lignin content on the mechanical properties of the developed lignin composite materials, tensile testing of the materials with varying lignin content from 0 wt.% to 40 wt.% was carried out. In **Figure** **4**a–c, the influence of the D‐OSL content on the Young's modulus, ultimate strength and strain at break are shown based on the individual tensile curves of each material in Figure 4d. Especially the stiffness of the material can be drastically improved by integration of lignin shown by an increase in Young's Modulus by a factor of 2.0, 4.0, 4.5, and 15.5 for lignin contents of 10 wt.%, 20 wt.%, 30 wt.% and 40 wt.%, respectively. Considering the rather large standard deviations, the ultimate strength of the materials reached the maximum of 1.8 MPa for a lignin content of 20 wt.% and kept nearly constant up to 40 wt.% demonstrating the strengthening effect of lignin filler. The strain at break showed its maximum of 5.8% at a lignin content of 10 wt.% and decreases for higher lignin content. This decrease in flexibility for high lignin content is in accordance with the increased stiffness caused by the rigid behavior of lignin. Both ultimate strength and strain at break could be significantly improved by integration of lignin by factor of up to 3.0 and 1.4, respectively. The impact properties of the materials are presented in Figure S6 (Supporting Information) confirming the improved toughness for a lignin content up to 20 wt.% and a reduced toughness for higher content due to the increased stiffness and rigidity. In conclusion, the integration of lignin at high content led to increased stiffness and strength and reduced flexibility. The resulting stiffness (308 MPa) of our lignin material is comparable to common polymer materials such as low‐density polyethylene (150–200 MPa),^[^ ^41^, ^42^ ^]^ polytetrafluoroethylene (500–600 MPa),^[^ ^43^, ^44^ ^]^ and polycaprolactone (190 MPa)^[^ ^45^ ^]^ providing suitable mechanical properties for various applications. In a similar composite of untreated lignin in a methacrylate resin, an increase in stiffness of 37% was observed for lignin content of 10–15 wt.%.^[^ ^46^ ^]^ This shows the same trend of increasing stiffness with higher lignin content as our materials. However, as the resin was limited in lignin content to 15 wt.% due to the strong absorption properties of lignin avoiding the UV curing of materials with higher content, the influence of higher lignin loading could not be investigated. In addition to mechanical performance, also optical properties, and wetting behavior of materials play an important role for their range of applications. To analyze the influence of the lignin content on the optical properties, UV/Vis transmission of the lignin materials was tested. In Figure 4e, the transmission spectra of 40 µm thin films of the lignin materials containing 0 wt.% to 40 wt.% of decolorized lignin are shown. As the reference material DL‐0 needs addition of Tinuvin as UV absorber for the 3D printing process, the optical properties of the material were analyzed with and without Tinuvin. It can be clearly seen that the addition of Tinuvin shifted the transmission limit from 300 nm to 390 nm. Since the decolorized lignin acts as an UV absorber no additional absorber was needed for 3D printing and optical properties were analyzed according to the resin composition in Table 1. The transmission spectra in Figure 4e show that with higher lignin content the transmission of UV and blue light decreased, while all materials show high transmission for green and red light. Thus, optical properties can be controlled by the lignin content resulting in materials with higher transparency for low lignin content or strong UV and blue light filter properties for high lignin content. As materials require chemical resistance and structural integrity toward water and ethanol for various applications such as outdoor devices or bioanalytical microsystems, the swelling behavior of the lignin composites was investigated. The influence of the lignin content on the swelling behavior was analyzed by immersing the samples into water and ethanol and testing their gain in weight. As can be seen in Figure 4f, the swelling in water of all materials is less than 2.5% in relation to the dry weight of the samples. There is only a small influence of the lignin content on the swelling behavior of the materials observable. While the reference material swelled by 1.6% over 24 h, the DL‐40 material with 40 wt.% lignin content swelled by 2.2%. Even in ethanol the lignin did not affect the swelling behavior as all composite materials with lignin content from 0 wt.% to 40 wt.% showed a similar swelling behavior in ethanol of 7.0% to 8.9% (see Figure S7a, Supporting Information). The ethanol used for the swelling experiment did not change its color, indicating that there was no diffusion of lignin content from the material of the composite (Figure S7b, Supporting Information). As the incorporation of lignin did not show any significant influence on the swelling behavior in water or ethanol, the lignin materials can be used for all applications in water and ethanol even at high filler content.

## 3 Conclusion

In this work, biocomposite materials for high‐resolution vat polymerization 3D printing with exceptionally high lignin contents of up to 40 wt.% were developed. To achieve this, lignin was decolorized by UV irradiation in THF using a low‐energy UV LED array of 14.4 W for 12 h. Besides the reuse of the THF for many process cycles, our decolorization process saved 99% of the energy and improved the decolorization degree from 65% to 82% compared to a previously reported process. To integrate the decolorized lignin in a 3D printable resin, a matrix composition of bio‐based tetrahydrofurfuryl acrylate and pentaerythritol tetraacrylate with high solubility for decolorized lignin was developed. By optimization of printing parameters such as exposure time according to the reactivity and absorption quality of the individual resins, the lignin composite materials with lignin content up to 40 wt.% could be 3D printed in high‐resolution enabling the fabrication of small, complex structures with feature sizes down to 250 µm. The development of composite materials with high lignin content does not only help to fabricate materials with potentially high sustainability, but also allows for the production of high‐performance materials with improved mechanical properties. By tensile testing it was shown that the integration of lignin of 40 wt.% increased the stiffness of the material by a factor of 15. Moreover, enhanced ultimate strength by a factor of 3.0 and flexibility by a factor of 1.4 were shown for lignin contents of 20 wt.% and 10 wt.%, respectively. The combination of improved mechanical properties and increased sustainability of the lignin composite materials with the ability for high‐precision structuring by vat polymerization 3D printing reduces the footprint of high‐resolution printing and enables numerous applications.

## 4 Experimental Section

### Materials

Pyridine (≥99%), acetic acid anhydride, tetrahydrofuran (THF), diethyl ether, methanol, acetone, toluene, and isopropanol were purchased from Carl Roth GmbH. Tetrahydrofurfuryl acrylate (THFA), pentaerythritol tetraacrylate (PETA), triethylamine, and diphenyl(2,4,6‐trimethylbenzoyl) phosphine oxide (TPO) were supplied by Sigma–Aldrich. 3‐(Trimethoxysilyl)propyl methacrylate was sourced from abcr. 2‐(5‐Chloro‐2H‐benzotriazol‐2‐yl)−4‐methyl‐6‐di‐tert‐butylphenol (Tinuvin 326) was purchased from BASF. Organosolv lignin (OSL), derived from a pulping process in ethanol/water (50:50 w:w) with 1 wt.% H_2_SO_4_ at 170 °C, followed by precipitation in water, was generously provided by the Fraunhofer Center for Chemical‐Biotechnological Processes (Leuna, Germany).^[^ ^47^ ^]^ All chemicals were used as received without any further purification.

### Acetylation of OSL

Acetylated organosolv lignin (A‐OSL) was synthesized by acetylation of OSL following an established literature procedure with minor adaption.^[^ ^32^ ^]^ Previous to acetylation, OSL was dried under vacuum (50 mbar) at 70 °C overnight. The dried OSL (18 g) was mixed with acetic anhydride (54 mL, 0.57 mol) and pyridine (180 mL, 2.23 mol) in a round bottom flask. The mixture was stirred with 1400 rpm for 72 h at room temperature. Subsequently, the reaction mixture was added dropwise into cold water. The resulting precipitate was filtered‐off and washed with cold water (3 × 80 ml). The final product A‐OSL was dried under vacuum (50 mbar) at 40 °C overnight.

### Decolorization of A‐OSL

Decolorized lignin (D‐OSL) was produced by UV irradiation of A‐OSL based on an optimized literature procedure.^[^ ^33^ ^]^ A‐OSL (1.25 g) was dissolved in THF (500 mL). The solution was then irradiated with UV light (365 nm, 14.4 W) for 12 h using an array of UV LEDs (15 × LEUVA35T01RL01 LED, LG Innotek, South Korea). The solution of D‐OSL in THF was then concentrated by a factor of ten by rotary evaporation to increase the efficiency of the precipitation process and also to recycle the THF. The concentrated D‐OSL solution was then added dropwise into diethyl ether. The precipitated D‐OSL was filtered‐off, washed with diethyl ether and dried at room temperature. The diethyl ether was also recycled by rotary evaporation of the filtrate.

### Resin Preparation

The individual resins were prepared by mixing the components according to Table 1. The procedure is described as exemplary for sample DL‐40. In a solution of PETA (7.8 wt.%) in THFA (52.2 wt.%) TPO (2.0 wt.%) is dissolved by stirring at room temperature for 20 min. Subsequently, D‐OSL (40 wt.%) is added to the solution and stirred for 2 h at 40 °C to obtain a homogeneous solution. As DL‐0 does not contain any lignin acting as a UV absorber, Tinuvin 326 (1.0 wt.%) was dissolved in the resin as an absorber, additionally. The prepared resins were stored at room temperature and protected from light till being used for 3D printing.

### 3D Printing

Structuring of the lignin materials by 3D printing was conducted using an Asiga MAX X27 DLP printer (Asiga, Australia) with an UV LED wavelength of 385 nm, a pixel resolution of 27 µm. All materials were printed with a UV light intensity of 29.3 mWcm^−2^ and a layer thickness of 25 µm. To ensure a sufficient adhesion of the printed objects to the substrate, functionalized glass slides were stuck to the print head as substrate. For functionalization, the commercial glass slides (26 mm × 26 mm × 1 mm) were cleaned in methanol:HCl (1:1, v:v) for 45 min and functionalized in a solution of (3‐methacryloxypropyl)dimethylchlorosilane (100 mM) in toluene for 90 min. After 3D printing, the objects were rinsed with acetone to remove the residual resin.

### UV–vis Analysis

UV–vis measurements were carried out using a QE‐Pro spectrometer (Ocean Optics, USA) equipped with the DH‐2000‐BAL light source and a temperature‐controlled cuvette holder qpod 2eTM (Quantum Northwest, USA). Both liquid resins and cured materials were measured as thin films between two glass slides with a thickness of 40 µm, while the neat two glass slides served as blank. For measurement of the cured materials, the resins were placed between the two glass slides in a thickness of 40 µm and cured by UV irradiation (320–500 nm) for 3 min using a Lumatec Superlite S04 lamp.

### Mechanical Properties

Tensile testing of the developed lignin materials was done using a dynamic analyzer MCR 702 (Anton Paar, Austria) in tensile mode with an elongation rate of 0.02 mm s^−1^ and an initial length of 9.0 mm. The samples in the shape of dog bones with a total length of 13.0 mm, a width of 2.0 mm, and a thickness of 1.0 mm were fabricated vertically according to the DLP 3D printing method described above. Each material was measured three times to calculate the average value and standard deviation.

### Swelling Test

For swelling test, three samples of each material in the shape of coins with a diameter of 6 mm and height of 3 mm were printed. All samples were weighed in dry state. The samples were then immersed in water and taken out for weighing after 5, 10, 20, 60, 210, 300, 1080 and, 1440 min. Before weighing, the water on the surface was removed by a tissue. The same experiment was carried out in ethanol.

### Optical Microscopy

For optical analysis of the 3D printed objects and their printing resolution, a VHX‐6000 microscope (Keyence, Japan) with a 20–200 magnification lens was used.

## Conflict of Interest

The authors declare no conflict of interest.

## Supporting information

Supporting Information

## Data Availability

The data that support the findings of this study are available in the supplementary material of this article.
