# Supplementary material for: Decolorization of Lignin for High‐Resolution 3D Printing of High Lignin‐Content Composites
Source: Adv Sci (Weinh). 2024 Aug 13;11(39):2406311. doi: 10.1002/advs.202406311 (PMC11497040; doi:10.1002/advs.202406311)
Supplement: Supplementary file 1 — Supporting Information [file ADVS-11-2406311-s001.docx]

Supporting Information

Decolorization of Lignin for High-Resolution 3D Printing of High Lignin-Content Composites

David Böcherer, Ramin Montazeri, Yuanyuan Li, Silvio Tisato, Leonhard Hambitzer, Dorothea Helmer*

**Recyclability of THF**

**Figure S1**. ^1^H-NMR spectra (300 MHz, CDCl_3_) of THF to analyze recyclability of THF in decolorization process. NMR spectra before UV irradiation and after 12 h of UV irradiation and recycling show identical results proving that the THF is not affected by the decolorization process and can be recycled to allow for a more sustainable process.

**Optical properties of untreated organosolv lignin (OSL) resins**


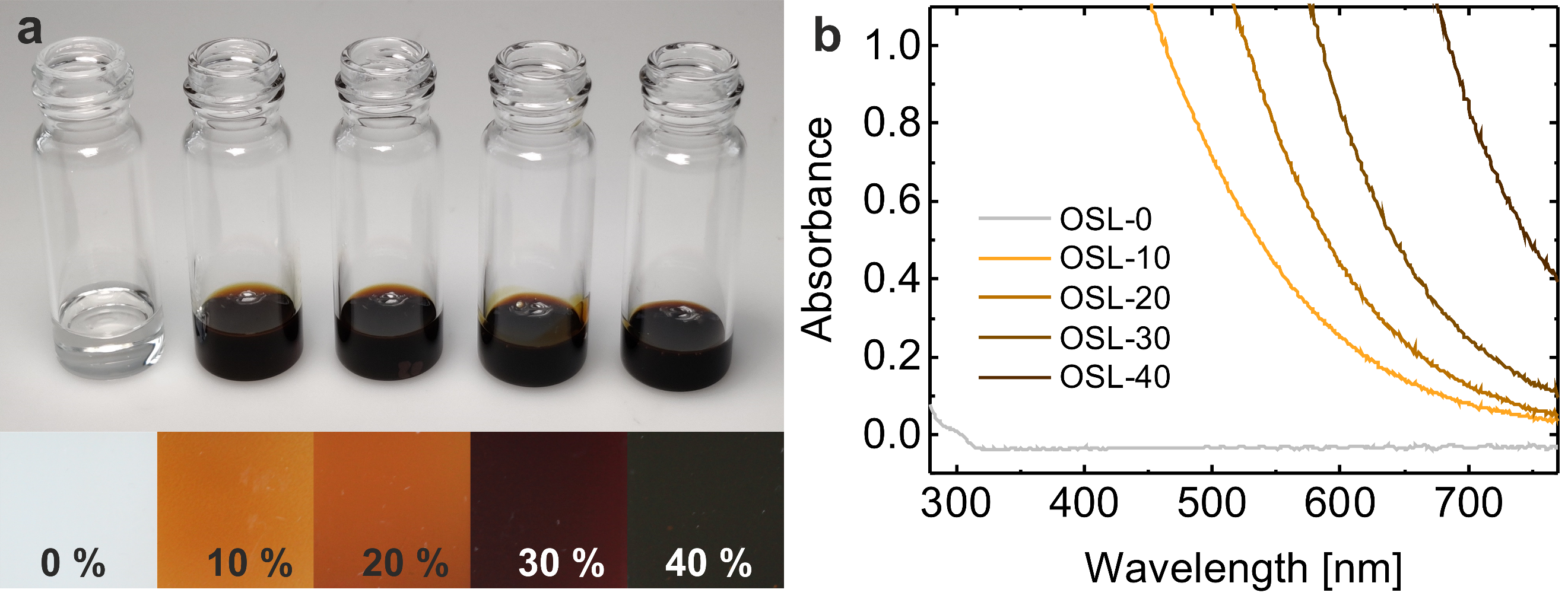


**Figure S2**. Optical properties of resins with untreated organosolv lignin (OSL) a) Optical appearance of the mixed resins with lignin contents from 0 wt% to 40 wt% in glass vials and as thin films. The bottom picture shows the thin liquid films of the resins of 40 µm thickness between two glass slides. Due to their strong absorption properties, these materials could not be cured by UV irradiation. b) Absorbance spectra of the individual resins with lignin contents from 0 wt% to 40 wt%. Absorbance was measured for the thin resin films shown in a).

**Viscosity of resin at elevated temperature**


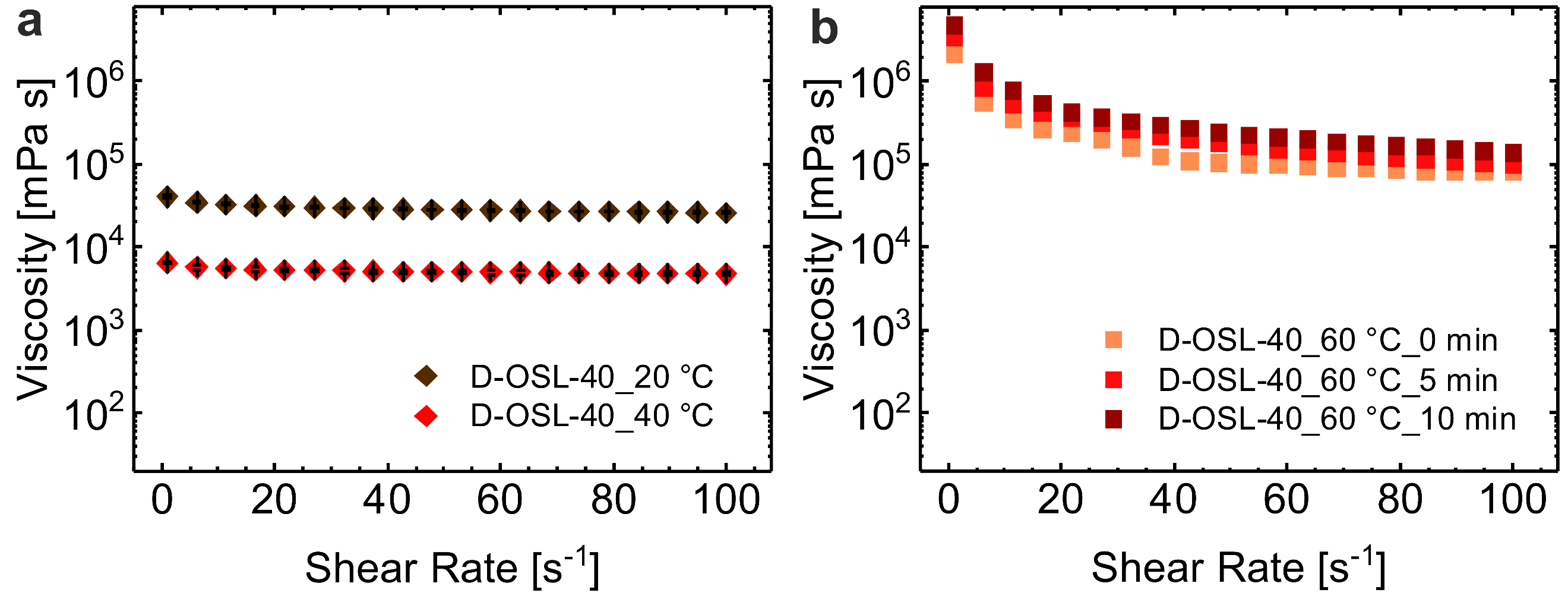


**Figure S3**. Shear rate-dependent viscosity measurements of the DL-40 resin with a lignin content of 40 wt% at different temperatures a) Viscosity of the DL-40 resin at 20 °C and 40 °C. Increasing the temperature from 20 °C to 40 °C decreases the viscosity by 82 % from 26013 mPa s to 4763 mPa s. The reduced viscosity at 40 °C enables a successful printing process of the material with high lignin content up to 40 wt%. b) Viscosity of the DL-40 resin at 60 °C after 0 min, 5 min and 10 min at elevated temperature. The viscosity increases significantly within a few minutes at 60 °C demonstrating the slow autopolymerization of the resin at high temperature.

**Curing depth calibration**


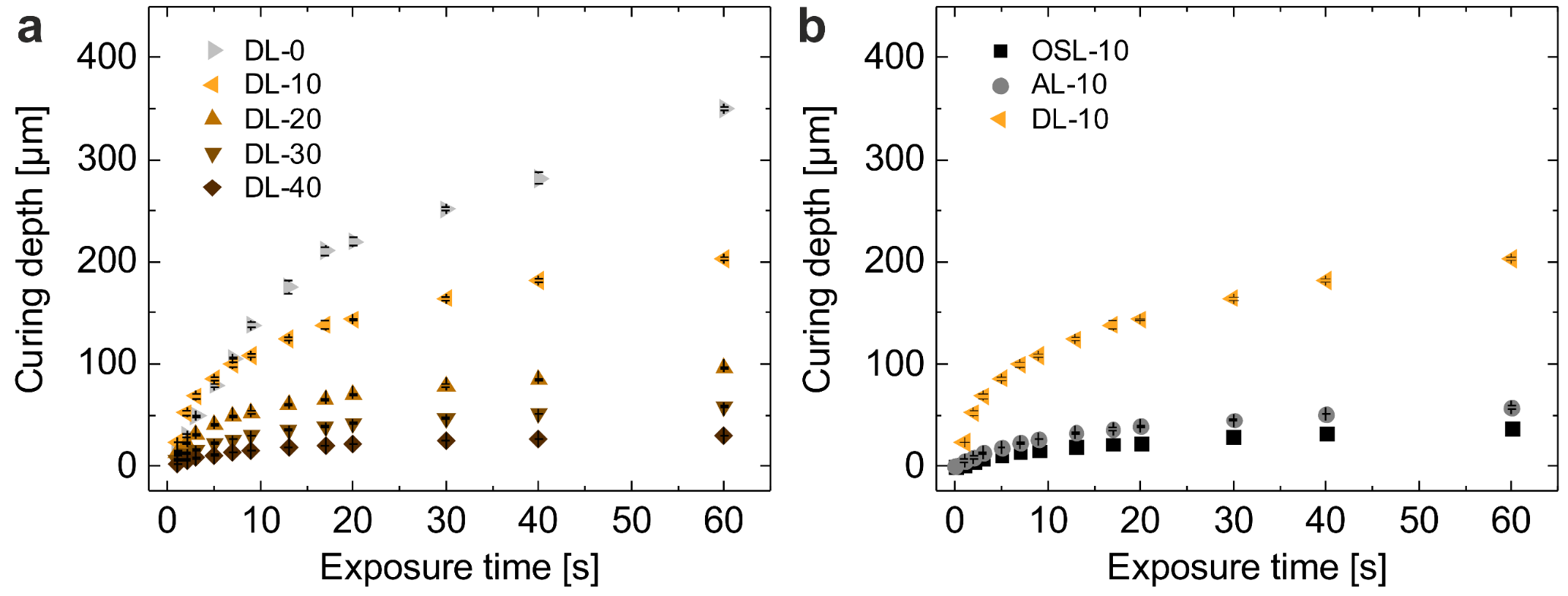


**Figure S4**. Curing depth calibration curves of the resins to analyze the influence of lignin content and lignin type on the curing properties by 3D printing process. a) Curing depth of resins with decolorized lignin from 0 wt% to 40 wt% depending on exposure time. The curing depth is significantly reduced from 350 µm to 35 µm by addition of 40 wt% of decolorized lignin. b) Curing depth of resins containing 10 wt% of pristine organosolv lignin (OSL), acetylated lignin (AL) or decolorized lignin (DL). It can be clearly seen that curing depth of the lignin resin is slightly improved by acetylation of OSL and drastically increased by decolorization. This proves that the decolorization process enables suitable light transmission properties for successful 3D printing of composite materials with high lignin content.

**Printing parameters**

**Table S1**. Optimized printing parameters for high-resolution printing of lignin composite materials with decolorized lignin content from 0 wt% to 40 wt%.

| Printing parameter | DL-0 | DL-10 | DL-20 | DL-30 | DL-40 |
| --- | --- | --- | --- | --- | --- |
| Exposure time [s] | 3 | 8 | 10 | 12 | 30 |
| Burn-in exposure time [s] | 16 | 57 | 249 | 300 | 360 |
| Temperature [°C] | 25 | 25 | 25 | 25 | 40 |
| Separation velocity [mm s^-1^] | 0.4 | 0.4 | 0.4 | 0.4 | 0.4 |
| Layer thickness [µm] | 25 | 25 | 25 | 25 | 25 |

**Printability of resins**

**
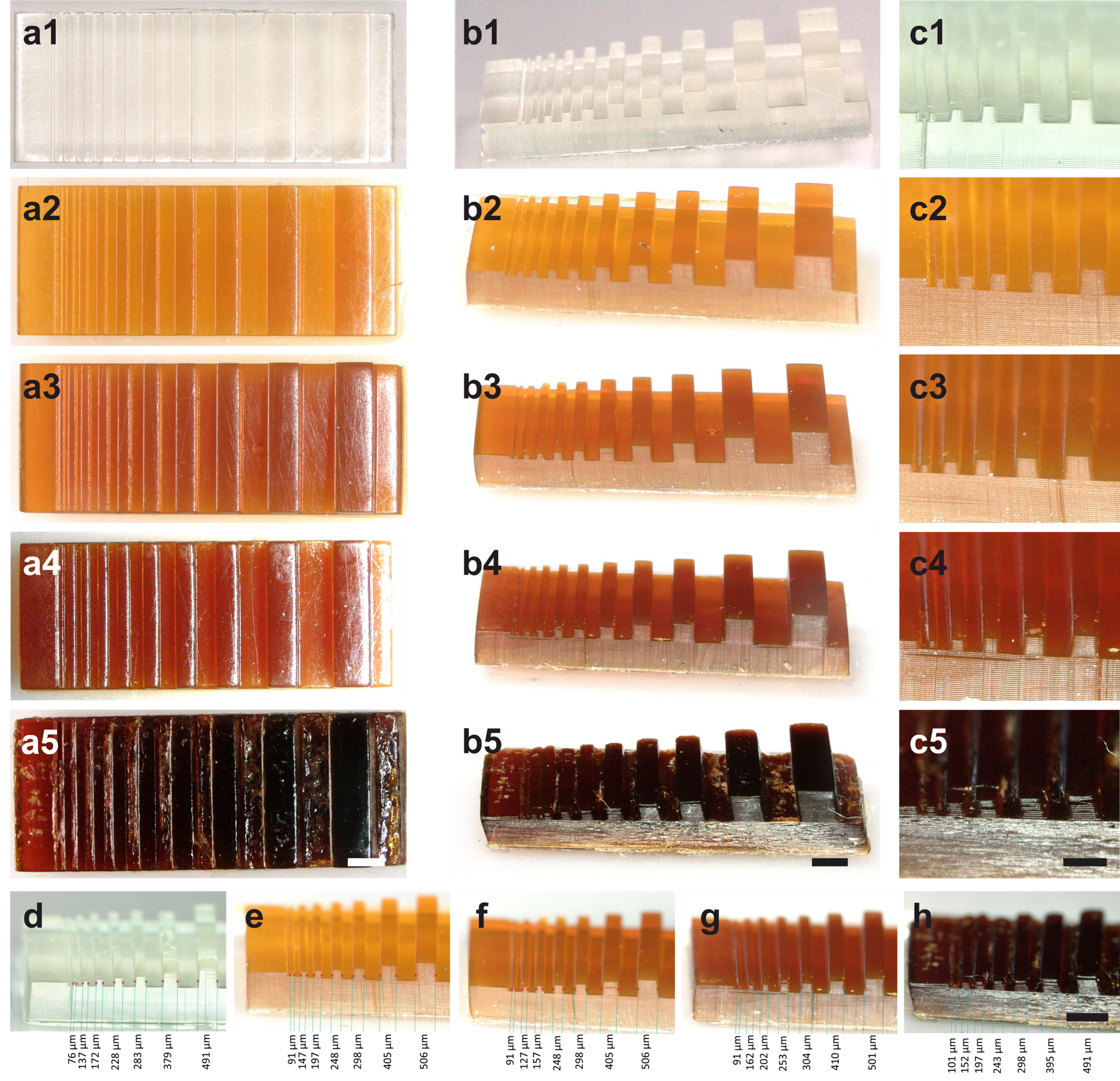
**

**Figure S5**. Microscopy images of printed structures for determination of the printability and resolution limit of each lignin material (1: DL-0, 2: DL-10, 3: DL-20, 4: DL-30, 5: DL-40). The design for the printability test consists of channels and bars in increasing size of 100, 150, 200, 250, 300, 400, 500, 600, 800, and 1000 µm. a) Top view of the printed lignin composites in the design for printability test demonstrating the good printability and high resolution down to 100 µm for all resins. Only the DL-40 material shows some small defects in surface structure caused by the high viscosity of the resin leading to reduced quality of the developing process. b) Side view of the printed design for each lignin material, demonstrating the precise width and height of the bars and the channels. c) Zoom-in on the smallest bars showing the high resolution of the prints down to 100 µm. d-h) Size measurements of the bars to determine the precision of the true structure compared to the design. All materials show a very high precision in design. Only the reference material DL-0 without any lignin shows an higher deviation than 10% caused by the weak mechanical properties and more distinct shrinkage of the material. Scale bars: a) 1 mm, b) 1 mm, c) 500 µm d-h) 1 mm.

**Impact properties**

**

**

**Figure S6**. Impact properties of lignin materials with lignin content from 0 wt% to 40 wt%. The charpy impact test was carried out by testing of six samples of each material with specimen dimensions of 20 mm × 5 mm × 2 mm and measuring gap of 1.5 mm. The results show the slight increase of the impact toughness with a lignin loading of 10 wt% and 20 wt%, while a higher loading significantly decreases the material toughness due to the strongly increased stiffness. Furthermore, the large standard deviation values need to be considered, which result from testing of the small sample sizes.

**Swelling in ethanol**


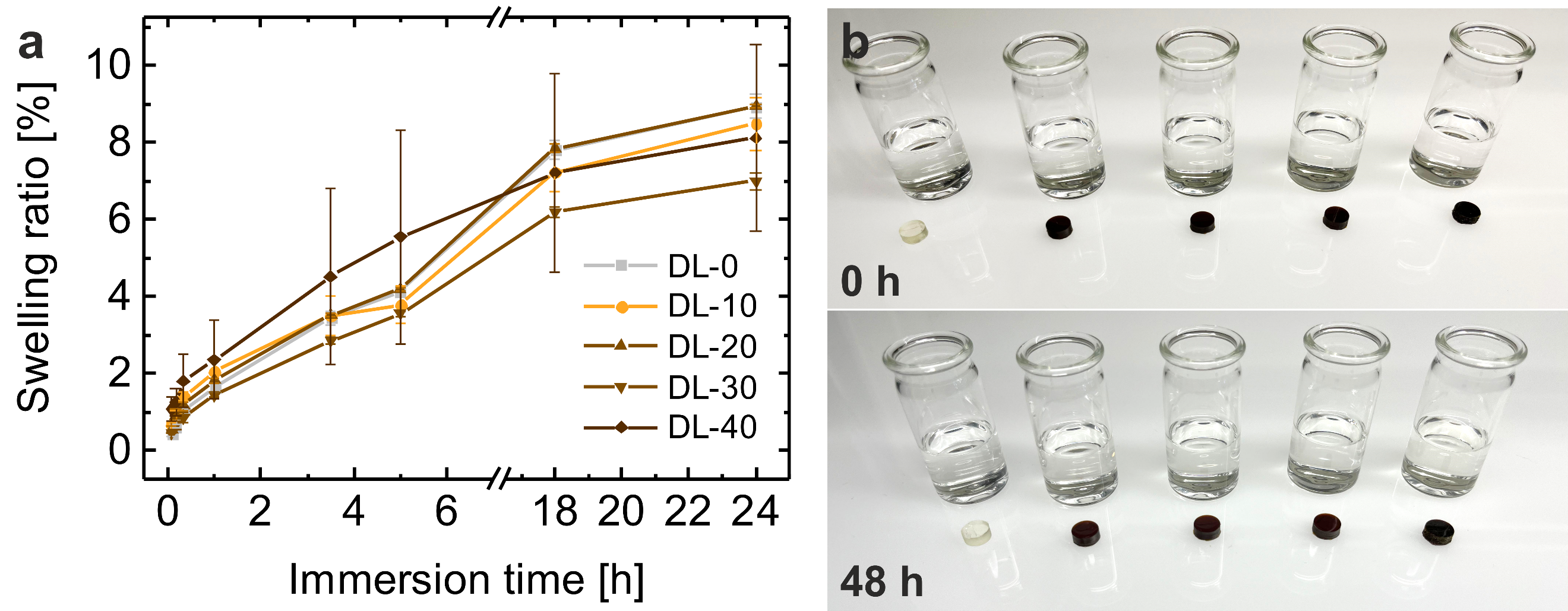


**Figure S7.** Swelling in ethanol of lignin materials with lignin content from 0 wt% to 40 wt%. a) Swelling test in ethanol of DL-0 to DL-40. All materials show a similar swelling behavior with a swelling degree of 7–9 % after 24 h indicating that the lignin filler does not significantly affect the swelling of the material. b) Pictures of the samples and the ethanol immersion solvent before (0 h) and after (48 h) the swelling test demonstrate that no coloration of the solvent occurs and thus indicate that no lignin was released from the samples into the ethanol.
